# Nomogram to predict the risk of acute kidney injury in patients with diabetic ketoacidosis: an analysis of the MIMIC-III database

**DOI:** 10.1186/s12902-021-00696-8

**Published:** 2021-03-04

**Authors:** Tingting Fan, Haosheng Wang, Jiaxin Wang, Wenrui Wang, Haifei Guan, Chuan Zhang

**Affiliations:** 1 Department of Endocrinology, Second Affiliated Hospital of Jilin University, Ziqiang Street 218, Changchun, 130041 Jilin China; 2 Department of Orthopedics, Second Affiliated Hospital of Jilin University, Changchun, China

**Keywords:** Diabetes mellitus, Diabetic ketoacidosis, Acute kidney injury, Nomogram

## Abstract

**Background:**

This study aimed to develop and validate a nomogram for predicting acute kidney injury (AKI) during the Intensive Care Unit (ICU) stay of patients with diabetic ketoacidosis (DKA).

**Methods:**

A total of 760 patients diagnosed with DKA from the Medical Information Mart for Intensive Care III (MIMIC-III) database were included and randomly divided into a training set (70%, *n* = 532) and a validation set (30%, *n* = 228). Clinical characteristics of the data set were utilized to establish a nomogram for the prediction of AKI during ICU stay. The least absolute shrinkage and selection operator (LASSO) regression was utilized to identified candidate predictors. Meanwhile, a multivariate logistic regression analysis was performed based on variables derived from LASSO regression, in which variables with *P* < 0.1 were included in the final model. Then, a nomogram was constructed applying these significant risk predictors based on a multivariate logistic regression model. The discriminatory ability of the model was determined by illustrating a receiver operating curve (ROC) and calculating the area under the curve (AUC). Moreover, the calibration plot and Hosmer-Lemeshow goodness-of-fit test (HL test) were conducted to evaluate the performance of our newly bullied nomogram. Decision curve analysis (DCA) was performed to evaluate the clinical net benefit.

**Results:**

A multivariable model that included type 2 diabetes mellitus (T2DM), microangiopathy, history of congestive heart failure (CHF), history of hypertension, diastolic blood pressure (DBP), urine output, Glasgow coma scale (GCS), and respiratory rate (RR) was represented as the nomogram. The predictive model demonstrated satisfied discrimination with an AUC of 0.747 (95% CI, 0.706–0.789) in the training dataset, and 0.712 (95% CI, 0.642–0.782) in the validation set. The nomogram showed well-calibrated according to the calibration plot and HL test (*P* > 0.05). DCA showed that our model was clinically useful.

**Conclusion:**

The nomogram predicted model for predicting AKI in patients with DKA was constructed. This predicted model can help clinical physicians to identify the patients with high risk earlier and prevent the occurrence of AKI and intervene timely to improve prognosis.

**Supplementary Information:**

The online version contains supplementary material available at 10.1186/s12902-021-00696-8.

## Background

Diabetic ketoacidosis (DKA) is a life-threatening metabolic complication of diabetes mellitus (DM), resulting from significant insulin deficiency and increased concentration of counterregulatory hormones [1]. Insulinopenia promotes the breakdown of triglycerides to free fatty acids (FFAs) by accelerating hormone-sensitive lipase (HSL). The FFAs are then oxidized to ketone bodies in the liver, the evaluation of FFAs exacerbates insulin resistance and hyperglycemia. Then glucose-induced osmotic polyuria and sometimes emesis incurred volume depletion, which generate three main characteristics--hyperglycemia, ketogenesis, and metabolic acidosis [2]. A retrospective study of 8533 patients with DKA in Australia showed that the prevalence of DKA admitting to the intensive care unit (ICU) has increased 5-fold over the last decade [3].

Acute kidney injury (AKI) is a frequent complication among hospitalized patients, associated with high mortality and morbidity, especially in critically ill patients [4]. The disease occurs in approximately 30–50% of ICU patients [5]. AKI is characterized by sudden worsening renal function and decreasing urine output, which leads to electrolyte and acid-base metabolic disorders, volume overload, and negative effects of these disturbances on other organs system [6]. Renal ischemia/reperfusion (I/R) injury is a frequent cause of AKI [7]. Glucose-induced dehydration is the main risk factor of AKI in DKA patients [8]. Junzhe Chen et al. reported 98 patients (54.75%) diagnosed as AKI among 179 DKA patients [9]. Early identification and management can decrease the AKI rate and delay its progression to the severe stage [10]. Therefore, it is necessary to assess the risk of suffering AKI in DKA patients given its seriousness.

Several risk factors of developing AKI for DKA patients have been investigated over the past decade, including older age, increased glucose, serum uric acid, white blood cell count (WBC), and hyperchloremia, heart rate (HR); decreased pH, serum albumin, bicarbonate, sodium; combined with coma on admission and preexisting chronic kidney diseases (CKD) [8, 9, 11]. There are no currently reliable and robust predicted models available to identify high-risk patients to develop AKI based on these factors.

A nomogram provides a user-friendly graphical tool to calculate the possibility of a noteworthy clinical event for each individual, which is comprehensible for patients in doctor-patient communication [12]. This study developed and verified a nomogram model to predict the morbidity of AKI during ICU stay in the DKA population, based on variables of the routine lab from the Medical Information Mart for Intensive Care III (MIMIC-III) database.

## Methods

### Data source and pre-processing

The MIMIC-III Database, a multiparameter critical care database open to the public at the Massachusetts Institute of Technology (MA, USA), was used [13]. The National Institutes of Health’s web-based course was completed and the certification (researcher certificate number: 9168028) was acquired. Data from the MIMIC-III database was collected using structured query language (SQL) software, the code of this process was demonstrated in the Supplementary Materials.

### Study population

We extracted the hadm id identifiers of 874 patients with DKA from the MIMIC-III database using the ICD-9 diagnostic code. Only the records of the first ICU stay were maintained for patients admitted to the ICU more than once during a single hospitalization; a total of 863 cases were obtained. Patients with CKD (stage 5) were excluded (*n* = 90). There were 13 patients excluded, whose missing value was > 20%. Eventually, 760 patients were included in the study. The training set (70%, *n* = 532) and validation set (30%, *n* = 228) were randomly assigned from the total cases.

### Clinic variables and definition

The following variables were extracted: Demographics, vital signs, laboratory tests, complications and comorbidities, scoring systems, and other variables. All data were collected within 24 h of ICU admission (Table 1). The demographics and vital signs included age, gender, weight, ethnicity, temperature, HR, respiratory rate (RR), systolic blood pressure (SBP), and diastolic blood pressure (DBP). Complications were as follows: Microangiopathy (diabetic nephropathy, diabetic retinopathy, and diabetic peripheral neuropathy), macroangiopathy (coronary heart disease, cerebral atherosclerosis, peripheral). Comorbidities contained preexisting CKD, urinary tract infection (UTI), pneumonia, liver disease, history of hypertension, history of congestive heart failure (CHF). The laboratory test included bicarbonate, WBC, hemoglobin, neutrophil granulocyte, blood platelet, sodium, chloride, blood urea nitrogen (BUN), serum creatinine (Scr), estimated glomerular filtration rate (eGFR), potassium, blood glucose, anion gap (AG), total osmotic pressure. Scoring systems included simplified acute physiology score II (SAPS II), sequential organ failure assessment (SOFA) score, oxford acute severity of illness score (OASIS), and Glasgow coma scale (GCS). Other collected data included DM type [type 1 diabetes mellitus (T1DM) and type 2 diabetes mellitus (T2DM)], infusion volume, urine output, use of NaHCO_3_, use of mechanical ventilation, hospital length of stay (HLOS), hospital mortality. Variables with a missing value > 20% were excluded (Ratio of missing data for excluded variables: serum uric acid 97%, C-reactive protein 96%, height 70%, urine protein 69%, glycated hemoglobin 67%, albumin 50%, PO_2_ 39%, PCO_2_ 0.39, PH 39%, lactate 28%, urine ketone 25%). A diagnosis of AKI during ICU stay was made when meeting KDIGO criteria [14]: [Scr increased by≥0.3 mg/dl within 48 h, or increase to≥1.5 fold from baseline within the prior 7 days, or urine volume < 0.5 ml/kg/h for 6 h or more]. The individuals’ baseline of Scr level was evaluated according to KDIGO criteria.

**Table 1:** Characteristic at baseline between AKI and non-AKI group

Variable	Total (*n* = 760)	Non-AKI (*n* = 446)	AKI (*n* = 314)	*P* value
Age, years	46.1 [33.7, 57.4]	42.7 [29.8, 55.9]	48.4 [36.7, 60.5]	< 0.001
Gender (Female)	438 (57.6)	250 (56.1)	188 (59.9)	0.330
Weight, Kg	72.7 [63.2, 82.9]	71.9 [63.5, 81.8]	74.2 [62.1, 84.9]	0.205
Ethnicity				0.19
Caucasian	429 (56.4)	252 (56.5)	177 (56.4)	
African-American	228 (30.0)	125 (28.0)	103 (32.8)	
Hispanic-American	35 (4.6)	25 (5.6)	10 (3.2)	
Other	68 (8.9)	44 (9.9)	24 (7.6)	
DM type				0.012
T1DM	525 (69.2)	324 (72.8)	201 (64.0)	
T2DM	234 (30.8)	121 (27.2)	113 (36.0)	
Temperature, °C	37.3 [37.0, 37.7]	37.3 [37.0, 37.6]	37.4 [37.0, 37.7]	0.010
HR, beats/min	108.0 [97.0, 120.0]	109.0 [99.0, 120.0]	107.0 [96.0, 119.0]	0.190
RR, breaths/min	25.4 [22.0, 29.0]	25.0 [22.0, 28.0]	26.0 [23.0, 30.0]	0.004
SBP, mmHg	98.0 [89.0, 108.0]	98.0 [90.0, 108.0]	97.0 [86.0, 108.0]	0.072
DBP, mmHg	46.0 [39.0, 54.0]	48.0 [41.0, 55.0]	44.5 [37.0, 52.0]	< 0.001
Microangiopathy	266 (35.0)	131 (29.4)	135 (43.0)	< 0.001
Macroangiopathy	137 (18.0)	60 (13.5)	77 (24.5)	< 0.001
Preexisting CKD	93 (12.2)	38 (8.5)	55 (17.5)	< 0.001
UTI	92 (12.1)	43 (9.6)	49 (15.6)	0.017
Pneumonia	48 (6.3)	19 (4.3)	29 (9.2)	0.006
Liver disease	60 (7.9)	30 (6.7)	30 (9.6)	0.173
History of hypertension	80 (10.5)	33 (7.4)	47 (15.0)	0.001
History of CHF	65 (8.6)	22 (4.9)	43 (13.7)	< 0.001
Bicarbonate, mEq/L	14.0 [9.0, 18.0]	13.0 [8.0, 18.0]	15.0 [10.0, 19.0]	< 0.001
WBC, K/uL	13.6 [9.9, 17.7]	13.3 [9.6, 17.6]	13.8 [10.5, 18.1]	0.177
Neutrophil granulocyte, %	83.0 [75.0, 88.3]	83.0 [75.6, 88.2]	83.0 [74.3, 88.5]	0.912
Platelets, K/uL	299.0 [236.0, 377.0]	299.0 [240.2, 369.8]	300.5 [232.0, 384.8]	0.902
Hemoglobin, g/dl	12.8 [11.4, 14.5]	13.3 [11.8, 14.8]	12.1 [10.8, 13.6]	< 0.001
Sodium, mEq/L	141.0 [138.0, 144.0]	140.0 [138.0, 144.0]	141.0 [138.0, 145.0]	0.036
Chloride, mEq/L	111.0 [107.0, 115.0]	111.0 [107.0, 114.0]	111.0 [107.0, 115.0]	0.859
AG	21.2 [17.6, 24.7]	21.4 [17.8, 25.2]	20.3 [17.2, 24.1]	0.029
Total osmotic pressure	319.7 [308.7, 334.8]	318.5 [308.1, 333.5]	322.0 [310.5, 336.0]	0.059
BUN, mg/dl	27.0 [17.0, 42.0]	24.0 [16.0, 37.8]	34.0 [19.0, 49.0]	< 0.001
Potassium, mEq/L	4.3 [3.8, 4.9]	4.3 [3.8, 5.0]	4.2 [3.8, 4.8]	0.353
Blood glucose, mg/dl	306.0 [164.5, 506.2]	331.5 [175.5, 525.8]	265.5 [153.2, 498.5]	0.044
SAPSII	27.0 [20.0, 36.0]	24.0 [19.0, 32.0]	30.0 [23.0, 41.0]	< 0.001
OASIS	25.0 [21.0, 31.0]	24.0 [21.0, 28.0]	28.0 [23.0, 34.0]	< 0.001
SOFA	2.0 [1.0, 4.0]	2.0 [1.0, 3.0]	3.0 [2.0, 5.0]	< 0.001
GCS	15.0 [14.0, 15.0]	15.0 [14.0, 15.0]	15.0 [14.0, 15.0]	< 0.001
Infusion volume, ml	1000.0 [0.0, 2750.5]	1000.0 [0.0, 2759.0]	1000.0 [0.0, 2733.8]	0.717
Urine output, ml	2071.6 [1396.8, 2959.2]	2200.0 [1543.2, 3217.2]	1790.0 [1148.5, 2670.0]	< 0.001
eGFR	98.2 [62.2, 123.0]	103.4 [71.8, 124.2]	86.0 [45.9, 118.3]	< 0.001
Use of NaHCO_3_	72 (9.5)	25 (5.6)	47 (15.0)	< 0.001
Mechanical ventilation	91 (12.0)	18 (4.0)	73 (23.2)	< 0.001
HLOS, days	4.3 [2.8, 7.4]	3.5 [2.3, 5.4]	6.4 [3.9, 10.4]	< 0.001
Hospital mortality	17 (2.2)	4 (0.9)	13 (4.1)	0.005

Abbreviations: *AKI* Acute kidney injury, *DM* Diabetic mellitus, *T1DM* Type 1 diabetic mellitus, *T2DM* Type 2 diabetic mellitus, *HR* Heart rate, *RR* Respiratory rate, *SBP* Systolic blood pressure, *DBP* Diastolic blood pressure, *CKD* Chronic kidney diseases, *UTI* Urinary tract infection, *CHF* Congestive heart failure, *WBC* White blood cell, *AG* Anion gap, *BUN* Blood urea nitrogen, *SAPSII* Simplified acute physiology score II, *OASIS* Oxford acute severity of illness score, *SOFA* Sequential organ failure assessment, *GCS* Glasgow coma scale, *eGFR* Estimated glomerular filtration rate, *HLOS* Hospital length of stay

Formula: AG = (Na^+^+K^+^) - (Cl^−^-HCO_3_^−^); Total osmotic pressure = 2(Na^+^+K^+^) + urea(mmol/l) + glucose (mmol/l)

### Statistical analysis

**Fig. 1:**
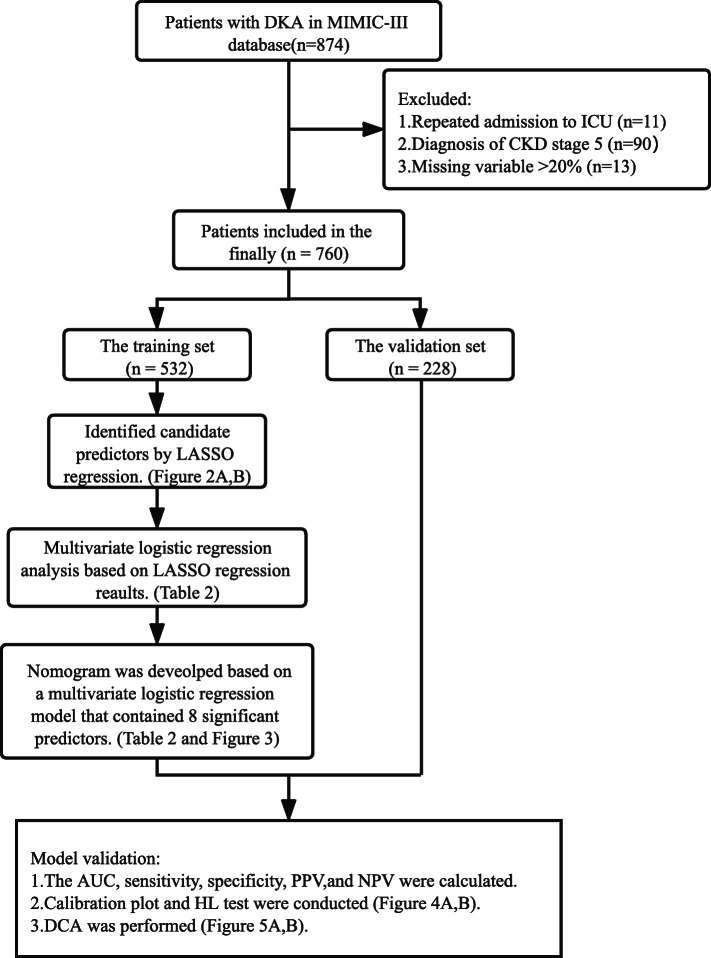
The flowchart of the study. DKA diabetic ketoacidosis, ICU intensive care unit, CKD chronic kidney diseases, LASSO least absolute shrinkage and selection operator, AUC area under the curve, PPV positive predictive value, NPV negative predictive value, HL test Hosmer-Lemeshow goodness-of-fit test, DCA Decision curve analysis

Wilcoxon’s rank-sum test, chi-square tests or Fisher’s exact test was conducted to compare the difference between the two groups; AKI versus non-AKI, and training set versus validation set. Missing data were filled up with nearest neighbor imputation algorithms [15]. Candidate features were conducted using univariate logistic analysis to assess the association between the variables and the endpoint. The least absolute shrinkage and selection operator (LASSO) regression was performed to screen the potential candidates. LASSO regression, using the “glmnet” package of R [16], is a linear regression that avoids overfitting by imposing a penalty on the magnitude of the model coefficients. Subsequently, a multivariate logistic regression analysis was performed based on variables derived from LASSO regression, in which variables with *P* < 0.1 were included in the final model. Ultimately, the nomogram was developed based on the final multivariate analysis model using the ‘rms’ package of R [17]. For the multivariate analysis model, one variable needs 20 samples of the endpoint at least [12]. The training set contained 228 positive endpoints, therefore there were at most 11 variables in our model. The area under the receiver operating curve (AUC), sensitivity, specificity, positive predictive value (PPV), and negative predictive value (NPV) were calculated to assess the apparent performance of the nomogram. A relatively corrected C-index (1000 bootstrap resamples) of the nomogram was also calculated in the training set. Meanwhile, the calibration plot and Hosmer-Lemeshow goodness-of-fit test (HL test) were used to evaluate the accuracy by comparing the nomogram. Decision curve analysis (DCA) was performed to assess the clinical usefulness of the predictive model [18]. All statistical analyses were performed using R statistical software (V.4.0.0). *P* < 0.05 was considered statistically significant. The research flowchart is shown in Fig. 1.

## Results

### Patient characteristics

Less than half of the 760 patients with DKA were diagnosed with AKI (*n* = 314, 41.3%). The incidence of AKI in stage 1, stage 2, and stage 3 were 44.9% (141/314), 36.6% (115/314), and 18.5% (58/314) respectively. The median age was 46.1 [IQR 33.7, 57.4] years, and 57.6% the of patients were women. Median SBP and DBP were 98 [IQR 89–108] mmHg, and 46 [IQR 39–54] mmHg.

The differences in patients’ characteristics between the AKI and the non-AKI groups are shown in Table 1. The hospital mortality and HOLS of patients in the AKI group (4.8% and 6.4 [IQR 3.9, 10.4] days) were significantly higher and longer than patients in the non-AKI group (4.1% and 3.5 [IQR 2.3, 5.4] days) (P<0.001). Compared with those without AKI, patients who suffered from AKI tend to have older years, higher temperature, bicarbonate, BUN and SAPSII, SOFA scores, and lower DBP, hemoglobin. Patients with AKI were also more likely to have hypertension, CHF, CKD, diabetic vascular complications, and T2DM. Interestingly, patients in the AKI group has a lower blood glucose level. The differences in patient characteristics between the training and validation sets were also compared (shown in Table S1). There were no differences between the two sets after comparing the two datasets.

### Characteristics selection and development of a nomogram

**Fig. 2:**
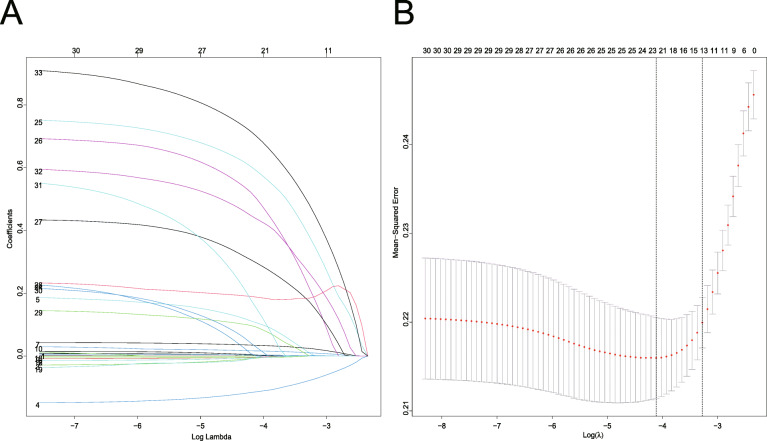
Demographic and clinical feature selection using the LASSO logistic regression model. **a** Tuning parameter (λ) selection using LASSO penalized logistic regression with 10-fold cross-validation. **b** LASSO coefficient profiles of the radiomic features. A coefficient profile plot was plotted versus the log (λ). Each colored line represents the coefficient of each feature

**Fig. 3:**
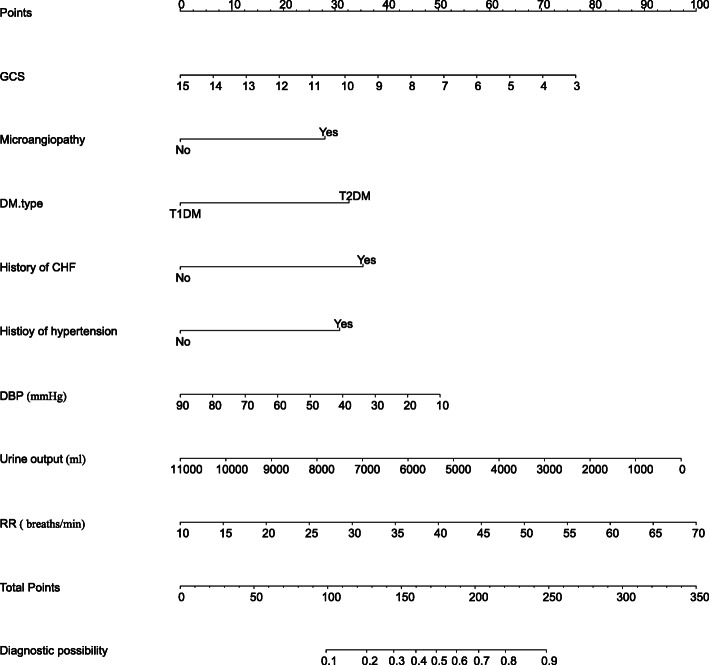
Nomogram to identify the risk of AKI after DKA, based on logistic regression analysis. To acquire the corresponding scores for each variable, draw a vertical line upward to the “Points” axis. Sum the score for all predictors and locate the final value on the “Total Points” axis. Draw a line straight down to the “Probability of AKI” axis to determine the risk of AKI. Abbreviations: AKI acute kidney injury, GCS Glasgow coma scale, DM diabetic mellitus, T1DM Type 1 diabetic mellitus, T2DM Type 2 diabetic mellitus, CHF congestive heart failure, DBP diastolic blood pressure, RR respiratory rate, CKD chronic kidney diseases, UTI urinary tract infection, BUN blood urea nitrogen

LASSO regression was conducted for 34 candidates, and 14 variables were selected (2.4:1 ratio) (Fig. 2a, b). The univariate logistic analysis results of the 34 candidates are shown in Table S2. The multivariable logistics analysis of the 14 variables are shown in Table 2. There were 8 predictors included in the final multivariable logistic model: including T2DM (OR: 2.61; 95% CI 1.68 to 4.11), microangiopathy (OR:2.28; 95% CI 1.49 to 3.52), preexisting CHF (OR: 2.83; 95% CI 1.38 to 6.13), history of hypertension (OR: 2.48; 95% CI 1.28 to 4.96), RR (OR: 1.05; 95% CI 1.02 to 1.09), urine output (OR: 1.00; 95% CI 1.00 to 1.00), GCS (OR: 0.83; 95% CI 0.73 to 0.93), DBP (OR: 0.98; 95% CI 0.97 to 1.00) (Table 2). In this model, a nomogram for predicting individuals’ probability of AKI during the ICU stay of patients with DKA was constructed (Fig. 3).

**Table 2:** Multivariate logistic regression model of AKI in the training set

Variables	Multivariable analysis base on LASSO regression result			Multivariable logistics model		
	β	OR (95%CI)	*P* value	β	OR (95%CI)	*P* value
GCS	−0.15	0.98 (0.97–1.00)	0.010	−0.19	0.83 (0.73–0.93)	< 0.05
Microangiopathy (yes)	0.75	2.11 (1.36–3.29)	< 0.001	0.82	2.28 (1.49–3.52)	< 0.001
DM type (T2DM)	0.84	2.31 (1.45–3.71)	< 0.001	0.96	2.62 (1.68–4.11)	< 0.001
History of CHF (yes)	0.94	2.55 (1.22–5.63)	0.016	1.04	2.83 (1.38–6.13)	< 0.05
History of hypertension (yes)	0.61	1.84 (0.59–5.86)	0.015	0.91	2.48 (1.28–4.96)	< 0.05
DBP, mmHg	−0.01	0.99 (0.97–1.00)	0.089	−0.02	0.98 (0.97–1.00)	< 0.05
Urine output, ml	0.00	1.00 (1.00–1.00)	< 0.001	−0.00	1.00 (1.00–1.00)	< 0.001
RR, breaths/min	0.05	1.05 (1.01–1.08)	0.008	0.05	1.05 (1.02–1.09)	< 0.05
Temperature, °C	0.19	1.21 (0.89–1.65)	0.222			
Preexisting-CKD (yes)	0.20	1.22 (0.42–3.66)	0.718			
Macroangiopathy (yes)	0.41	1.50 (0.90–2.51)	0.117			
UTI (Yes)	0.16	1.17 (0.65–2.13)	0.597			
BUN, mg/dl	0.00	1.00 (0.99–1.01)	0.472			
Bicarbonate, mEq/L	0.02	1.02 (0.99–1.06)	0.210			

Abbreviations: *AKI* Acute kidney injury, *LASSO* Least absolute shrinkage and selection operator, *β* Regression coefficient, *OR* Odds ratios, *GCS* Glasgow coma scale, *DM* Diabetic mellitus, *T2DM* Type 2 diabetic mellitus, *CHF* Congestive heart failure, *DBP* Diastolic blood pressure, *RR* Respiratory rate, *CKD* Chronic kidney diseases, *UTI* Urinary tract infection, *BUN* Blood urea nitrogen

### Apparent performance of the Nomogram

**Fig. 4:**
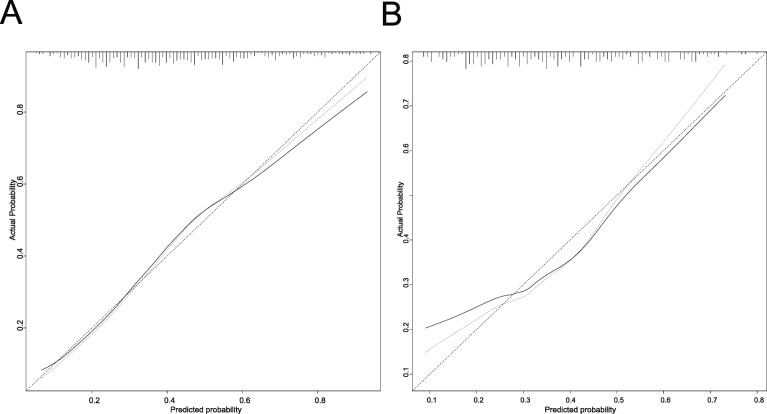
Calibration curves of the predicted nomogram in the training set (**a**) and validation set (**b**). The x-axis represents the predicted probability calculated by the nomogram, and the y-axis is the observed actual probability of AKI. The clinodiagonal represents a perfect prediction by an ideal model. The solid curve represents the initial cohort and the dotted curve is bias corrected by bootstrapping (B = 1000 repetitions), which demonstrates the performance of the predicted model. Results of the Hosmer-Lemeshow test demonstrate that the *P*-value of the training set (**a**) is 0.844 and the validation set (**b**) is 0.244, respectively

The AUC for the AKI nomogram was 0.747 (95% CI: 0.706–0.789) for the training set. The relatively corrected C-index was 0.733 after 1000 bootstrapping validation. In the validation dataset, the AUC also reached 0.712 (95% CI: 0.642–0.782). The optimal cutoff values for the AKI nomogram predicted probability was set at 34.1% in the training set and 38.5% in the validation set, according to the maximum of the Youden index. The sensitivity, specificity, PPV, and NPV were 83.8, 55.9, 58.8, 82.1% in train dataset, and 66.3, 69.7, 57.0, 77.3% in validation set. The calibration plot showed a good fitting degree of the nomogram for both cohorts (Fig. 4a, b). Additionally, the HL test of multivariable analysis demonstrated perfect consistency between the predicted and observed values (training set, χ2 = 4.885, *P* = 0.844; validation set, χ2 = 11.478, *P* = 0.244).

### Clinical practice

**Fig. 5:**
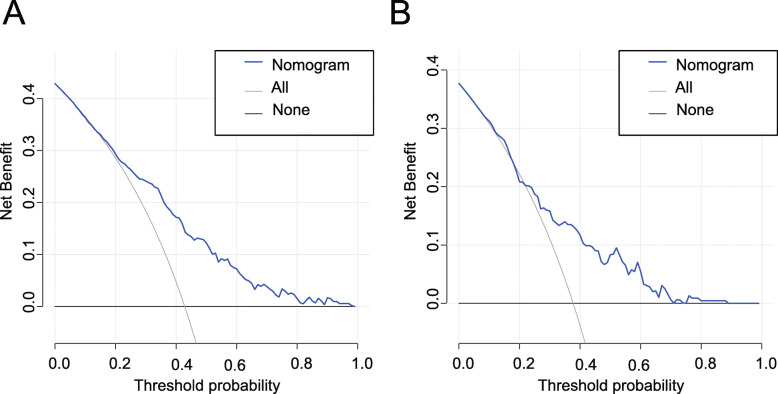
DCA of the nomogram in the training set (**a**) and the validation set (**b**). The horizontal line indicates no patients develop AKI, and the gray oblique line indicates patients develop AKI. The blue solid line represents the AKI risk nomogram. In DCA, the nomogram shows a more net benefit than full or no treatment across a threshold probability range. DCA decision curve analysis, AKI acute kidney injury

DCA for the AKI nomogram was conducted in both training and validation sets (Fig. 5a, b). The horizontal axis indicates that no one receives the intervention, the net benefit is 0. The oblique line indicates that all people received the intervention. When the predicted probability thresholds are set as 17–100% and 23–71% in the developing and validation cohort, the net benefit ranges 0–31% and 0–20%, respectively. The smaller the threshold, the net benefit.

## Discussion

This study focused on the short-term outcome of AKI after DKA by developing a nomogram model to investigate factors that could induce its occurrence using the routine information in ICU. Our study demonstrated that the incidence of AKI from DKA patients in our study is 41.3% according to 2012 KDIGO during the ICU stay. The incidence is slightly lower than is reported in previously published studies [8, 9, 11], but it is also higher than the general patients’ comparing with DKA patients. The HLOS, hospital mortality, and use of mechanical ventilation in the AKI group were much higher than the non-AKI group, which indicated adverse effects and a considerable economic burden for patients developing AKI. Therefore, it is essential to develop a nomogram for clinicians to identify patients at high risk and understand how the risk factors impact the outcome. The predictive nomogram incorporates 8 predictors: T2DM, microangiopathy, history of CHF, history of hypertension, DBP, urine output, GCS, and RR. The AUC of our model was 0.747 in the training set and 0.712 in the validation set, indicating that the performance of the model was satisfied. The calibration plot showed satisfied consistency between the actual and predicted. The DCA result demonstrated that applying this nomogram to predict AKI could benefit more than measures to treat all patients or not treat any patients.

Here we cite an example to show how to use the nomogram, assuming a DKA patient with T2DM, no history of CHF and hypertension, but with microangiopathy, his urine output was 2000 mL, RR was 25 breaths per minute, DBP was 50 mmHg, and GCS was 15. According to Fig. 3, the score corresponding to each parameter on the “Points” axis is obtained. The final score is calculated as the sum of points for all parameters [6 (GCS) + 28 (microangiopathy) + 32.5 (DM type) + 0 (history of hypertension) + 0 (history of CHF) + 25.5 (DBP) + 80 (urine output) + 10 (RR) =182]. This score corresponds to a risk of developing AKI during ICU stay of approximately 57%.

Our study comprehensively analyzed the relationship between diabetic chronic complications and DKA-AKI. There was a more frequent microvascular and macrovascular complication in patients with AKI group (*P* < 0.05), but macrovascular complication was not a significant variable in multivariate analysis. A previous study has reported that there is a reduction in the net capillary fluid absorption and mobilization of venous capacitance blood (capacitance response) in diabetes with microvascular complications, which is associated with increased risk of hemodynamic instability and reduced tolerance to hypovolemia [19]. Therefore, stricter liquid management should be performed for DKA patients with microvascular. Compared to T1DM, T2DM patients had a larger proportion of DKA-AKI (48.1% vs. 38.3%, *P* < 0.05). In the multivariate regression model, T2DM patients were associated with a more than 2.5-fold increase in the odds of DKA-AKI than T1DM patients. Patients with T2DM are mostly older and with a higher percentage of being overweight; increasing age [9] and obesity [20] are associated with AKI, which could be one explanation of our result. Univariable regression analysis showed that older age is significantly associated with AKI (OR = 1.01, P < 0.05), which had no correlation weight (OR = 1.01, *P* = 0.06). There was regrettably much missing data on patients’ height, so the body mass index was not calculated and could not assess the correlation between obesity with AKI. Besides, insulin resistance and characteristics of T2DM are frequently observed in severe patients with acute renal failure [21]. Treatment for DKA should distinguish between T1DM and T2DM patients due to differences in pathophysiology, and the increasing incidence of T2DM. Patients with a history of CHF have an increased incidence of AKI due to the low renal functional reserve. Low cardiac output or congestive state, as well as the influence of drugs, such as diuretics and angiotensin-converting enzyme inhibitors (ACEIs), are all related factors [22]. The OR for urine output was 0.99974; as the unit of this parameter was ml if a patient’s urine volume is 1000 mL, the OR becomes 0.77 (0.99974^1000^) for patients with no urination [23]. This result indicated that the lesser the urine volume at admission in ICU, the higher the risk for developing AKI. Urine output, in the clinical setting, is a common indicator for physicians to determine whether hemodynamics and infusion volume is appropriate or not. History of hypertension and lower DBP are closely related to a higher risk of AKI [24–26], which were selected as predictors by LASSO regression analyses in our model. Hypertension affects more than two-thirds of patients with T2DM [27]. Early antihypertensive therapy may further reduce renal perfusion and worsened renal outcome after AKI [28]. The decrease in blood pressure is a manifestation of hypovolemia, and SBP and DBP were significantly decreased in both groups of patients in this study cohort, which is in accord with the pathophysiological mechanism of DKA-capacity depletion due to dehydration [29, 30]. However there was no significant difference in SBP between the two groups, and DBP was significantly lower in the AKI group. Therefore, it remains to determine whether to continue antihypertensive treatment for DKA patients with hypertensives. Brain edema may occur under the combined action of multiple factors such as severe water loss, circulatory disorder, increased osmotic pressure, and brain cell hypoxia, causing central nervous dysfunction and different degrees of disturbance of consciousness [31, 32]. These pathological processes also contribute to the occurrence of AKI. Patients’ GCS reflect the severity of DKA and maybe the independent predictor of mortality at 1 year after ICU admission [33]. Therefore, lower GCS was associated with of high risk of AKI during ICU stay in our study. The RR was significantly higher in the AKI group than in the non-AKI group (26.0 [23.0, 30.0] vs 25.0 [22.0, 28.0]). Metabolic acidosis leads to hyperventilation and a decrease in CO_2_ concentration, preventing further decreases in pH and serum bicarbonate. As acidosis progresses, RR accelerates, and tidal volume increases, known as Kussmaul’s breath [34]. Microcirculatory disturbance due to acidosis and fluid loss caused by hyperventilation may explain RR as an independent risk factor for predicting AKI. Interestingly, we found lower serum bicarbonate in the non-AKI group in contrast to previous studies [11, 35]. The OR was 1.06 (*P* < 0.001), which paradoxically indicated that patients with mild DKA were at higher risk of AKI; differences in the study population may have lead to the conflicting finding. Infections, especially sepsis, is usually considered the most important risk factor for AKI; it is one of the commonest inducements of DKA [36, 37]. Patients in the AKI group seemed to be more likely to have pneumonia and urinary tract infection, but both of them were excluded from the final model after LASSO and multivariate analysis. This might be because there were only a few patients who have suffered infection at admission in our study. Besides, the blood glucose in the AKI group was lower than the non-AKI group, which in contrast to previous studies [8]. The use of insulin in the medicine department before admitting to ICU may explain this phenomenon. Studies have reported AKI nomogram in other settings, whose predictors were often associated with the primary disease. For instance, sepsis-induced AKI nomogram contained temperature as a risk factor [23], cardiac surgery-associated AKI predicted model included transfusion and cardiac arrhythmia as predictors [38], and contrast-induced nomogram considered heart rate and percutaneous coronary as predicted variables [39]. Although this study referred to some of these variables, DKA-induced AKI needs to consider the characteristics of diabetic patients. It is therefore highly desirable to develop a predictive model suitable for patients with DKA.

We first construct the nomogram to access the risk of AKI in patients who suffered DKA. Also, we found results contrary to previous studies, such as lower blood glucose and higher serum bicarbonate in patients with AKI, which provided a new problem for research to explore. However, the study had several limitations. First, these data were from a single institution spanning 2008 to 2012. Therefore, the model needs external validation from different medical Institutions. Second, because missing data is > 20% in the database, there is a lack of assessment of serum uric acid and urine protein, which is considered as independent risk factors in previous studies. Finally, the data of this study were collected within 24 h of ICU admission, in which the laboratory variables may have changed after treatment in the emergency department and general ward. Besides, the predicted model was constructed based on critically-ill DKA patients in ICU only, which may limit the nomogram’s application to a larger population. Therefore, the model may be more accurate and more generally applicable with the inclusion of new variables and patients in the general ward.

## Conclusions

It was identified that T2DM, microangiopathy, history of CHF, history of hypertension, DBP, urine output, GCS, and RR were predictive parameters for AKI induced by DKA. Additionally, a nomogram model was developed based on multiple logistics analyses with these predictors to predict AKI in patients with DKA. This model can help clinical physicians identify the patients with high risk earlier and to some extent prevent the occurrence of AKI.

## Supplementary Information

**Additional file 1: Table S1.** Characteristics of patients in the training and validation datasets.

**Additional file 2: Table S2.** Univariate logistic regression analysis.

**Additional file 3: **SQL code for data extraction.

## Abbreviations

AKI: Acute kidney injury
ICU: Intensive Care Unit
DKA: Diabetic ketoacidosis
LASSO regression: Least absolute shrinkage and selection operator regression
β: Regression coefficient
OR: Odds ratios
ROC: Receiver operating curve
AUC: Area under the curve
HL test: Hosmer-Lemeshow goodness-of-fit test
DCA: Decision curve analysis
T2DM: Type 2 diabetes mellitus
CHF: Congestive heart failure
DBP: Diastolic blood pressure
GCS: Glasgow coma scale
RR: Respiratory rate
DM: Diabetes mellitus
FFAs: Free fatty acids
HSL: Hormone-sensitive lipase
WBC: White blood cell count
HR: Heart rate
CKD: Chronic kidney diseases
SQL: Structured query language
SBP: Systolic blood pressure
UTI: Urinary tract infection
BUN: Blood urea nitrogen
Scr: Serum creatinine
eGFR: Estimated glomerular filtration rate
AG: Anion gap
SAPS II: Simplified acute physiology score II
SOFA: Sequential organ failure assessment
OASIS: Oxford acute severity of illness score
T1DM: Type 1 diabetes mellitus
HLOS: Hospital length of stay
PPV: Positive predictive value
NPV: Negative predictive value
ACEIs: Angiotensin-converting enzyme inhibitors

## References

[1] H D, X S, H L, L Z (2020). Association between red blood cell distribution width and mortality in diabetic ketoacidosis. J Int Med Res.

[2] M F, FJ P, GE U (2017). Management of Hyperglycemic Crises: diabetic ketoacidosis and hyperglycemic hyperosmolar state. Med Clin N Am.

[3] Venkatesh B, Pilcher D, Prins J, Bellomo R, Morgan TJ, Bailey M (2015). Incidence and outcome of adults with diabetic ketoacidosis admitted to ICUs in Australia and New Zealand. Critical care (London, England).

[4] Hoste EAJ, Bagshaw SM, Bellomo R, Cely CM, Colman R, Cruz DN (2015). Epidemiology of acute kidney injury in critically ill patients: the multinational AKI-EPI study. Intensive Care Med.

[5] Lewington A, Cerdá J, Mehta R (2013). Raising awareness of acute kidney injury: a global perspective of a silent killer. Kidney Int.

[6] Clec'h C, Darmon M, Lautrette A, Chemouni F, Azoulay E, Schwebel C (2012). Efficacy of renal replacement therapy in critically ill patients: a propensity analysis. Critical care (London, England).

[7] Bai J, Zhao J, Cui D, Wang F, Song Y, Cheng L (2018). Protective effect of hydroxysafflor yellow a against acute kidney injury via the TLR4/NF-κB signaling pathway. Sci Rep.

[8] Orban J-C, Maizière E-M, Ghaddab A, Van Obberghen E, Ichai C (2014). Incidence and characteristics of acute kidney injury in severe diabetic ketoacidosis. PLoS One.

[9] Chen J, Zeng H, Ouyang X, Zhu M, Huang Q, Yu W (2020). The incidence, risk factors, and long-term outcomes of acute kidney injury in hospitalized diabetic ketoacidosis patients. BMC Nephrol.

[10] Kashani K, Ronco C (2016). Acute kidney injury electronic alert for nephrologist: reactive versus proactive?. Blood Purif.

[11] Hursh BE, Ronsley R, Islam N, Mammen C, Panagiotopoulos C (2017). Acute kidney injury in children with type 1 diabetes hospitalized for diabetic ketoacidosis. JAMA Pediatr.

[12] Zhou Z-R, Wang W-W, Li Y, Jin K-R, Wang X-Y, Wang Z-W (2019). In-depth mining of clinical data: the construction of clinical prediction model with R. Ann Transl Med.

[13] Johnson AEW, Pollard TJ, Shen L, Lehman L-WH, Feng M, Ghassemi M (2016). MIMIC-III, a freely accessible critical care database. Sci Data.

[14] Kellum JA, Lameire N (2013). Diagnosis, evaluation, and management of acute kidney injury: a KDIGO summary (part 1). Crit Care (London, England).

[15] Beretta L, Santaniello A (2016). Nearest neighbor imputation algorithms: a critical evaluation. BMC Med Inform Decis Making.

[16] Friedman J, Hastie T, Tibshirani R (2010). Regularization paths for generalized linear models via coordinate descent. J Stat Softw.

[17] Harrell F, Lee K, Califf R, Pryor D, Rosati R (1984). Regression modelling strategies for improved prognostic prediction. Stat Med.

[18] Vickers A, Cronin A, Elkin E, Gonen M (2008). Extensions to decision curve analysis, a novel method for evaluating diagnostic tests, prediction models and molecular markers. BMC Med Inform Decis Making.

[19] Lindenberger M, Lindström T, Länne T (2013). Decreased circulatory response to hypovolemic stress in young women with type 1 diabetes. Diabetes Care.

[20] Martini A, Sfakianos JP, Paulucci DJ, Abaza R, Eun DD, Bhandari A (2019). Predicting acute kidney injury after robot-assisted partial nephrectomy: implications for patient selection and postoperative management. Urol Oncol.

[21] Basi S, Pupim LB, Simmons EM, Sezer MT, Shyr Y, Freedman S (2005). Insulin resistance in critically ill patients with acute renal failure. Am J Physiol Ren Physiol.

[22] Holgado JL, Lopez C, Fernandez A, Sauri I, Uso R, Trillo JL (2020). Acute kidney injury in heart failure: a population study. ESC Heart Failure.

[23] Deng F, Peng M, Li J, Chen Y, Zhang B, Zhao S (2020). Nomogram to predict the risk of septic acute kidney injury in the first 24 h of admission: an analysis of intensive care unit data. Ren Fail.

[24] Chen Z, McCulloch CE, Powe NR, Heung M, Saran R, Pavkov ME (2020). Exploring reasons for state-level variation in incidence of dialysis-requiring acute kidney injury (AKI-D) in the United States. BMC Nephrol.

[25] Huang Y, Wan C, Wu G (2020). Acute kidney injury after a stroke: a PRISMA-compliant meta-analysis. Brain Behav.

[26] Kane-Gill S, Sileanu F, Murugan R, Trietley G, Handler S, Kellum J (2015). Risk factors for acute kidney injury in older adults with critical illness: a retrospective cohort study. Am J Kidney Dis.

[27] Cloutier L, Lamarre-Cliche M (2018). Hypertension in adults with type 2 diabetes: a review of blood pressure measurement methods, targets and therapy. Can J Diabetes.

[28] Greite R, Derlin K, Hensen B, Thorenz A, Rong S, Chen R (2020). Early antihypertensive treatment and ischemia-induced acute kidney injury. Am J Physiol Ren Physiol.

[29] Umpierrez G, Korytkowski M (2016). Diabetic emergencies - ketoacidosis, hyperglycaemic hyperosmolar state and hypoglycaemia. Nat Rev Endocrinol.

[30] Infante B, Franzin R, Madio D, Calvaruso M, Maiorano A, Sangregorio F (2020). Molecular mechanisms of AKI in the elderly: from animal models to therapeutic intervention. J Clin Med.

[31] Nett S, Noble J, Levin D, Cvijanovich N, Vavilala M, Jarvis J (2014). Biomarkers and genetics of brain injury risk in diabetic ketoacidosis: a pilot study. J Pediatr Intens Care.

[32] Guisado R, Arieff AI (1975). Neurologic manifestations of diabetic comas: correlation with biochemical alterations in the brain. Metab Clin Exp.

[33] Ramaesh A (2016). Incidence and long-term outcomes of adult patients with diabetic ketoacidosis admitted to intensive care: a retrospective cohort study. J Intensive Care Soc.

[34] Gallo de Moraes A, Surani S (2019). Effects of diabetic ketoacidosis in the respiratory system. World J Diabetes.

[35] Kendrick J, Chonchol M, You Z, Jovanovic A. Lower serum bicarbonate is associated with an increased risk of acute kidney injury. J Nephrol. 2020. 10.1007/s40620-020-00747-8 32436182

[36] Chen JCY, Hu B, Frank RD, Kashani KB. Inpatient kidney function recovery among septic shock patients who initiated kidney replacement therapy in the hospital. Nephron. 2020:1–9. 10.1159/000507999 32575100

[37] Calliari LE, Almeida FJ, Noronha RM (2020). Infections in children with diabetes. J Pediatr.

[38] Guan C, Li C, Xu L, Zhen L, Zhang Y, Zhao L (2019). Risk factors of cardiac surgery-associated acute kidney injury: development and validation of a perioperative predictive nomogram. J Nephrol.

[39] Lei L, Xue Y, Guo Z, Liu B, He Y, Liu J (2020). Nomogram for contrast-induced acute kidney injury in patients with chronic kidney disease undergoing coronary angiography in China: a cohort study. BMJ Open.

