# Supplementary material for: Nomogram to predict the risk of acute kidney injury in patients with diabetic ketoacidosis: an analysis of the MIMIC-III database
Source: BMC Endocr Disord. 2021 Mar 4;21:37. doi: 10.1186/s12902-021-00696-8 (PMC7931351; doi:10.1186/s12902-021-00696-8)
Supplement: Supplementary file 1 — Additional file 1: Table S1. Characteristics of patients in the training and validation datasets. [file 12902_2021_696_MOESM1_ESM.docx]

**Table S1** Characteristics of patients in the training and validation datasets

| Variable | Training dataset  (n=532) | Validation dataset  (n=228) | P value |
| --- | --- | --- | --- |
| Age, years | 46.4 [34.1, 57.9] | 45.5 [32.8, 57.2] | 0.420 |
| Gender (Female) | 299 (56.2) | 139 (61.0) | 0.255 |
| Weight, Kg | 72.7 [63.4, 82.9] | 72.4 [62.3, 82.9] | 0.607 |
| Ethnicity |  |  | 0.069 |
| Caucasian | 301 (56.6) | 128 (56.1) |  |
| African-American | 167 (31.4) | 61 (26.8) |  |
| Hispanic-American | 18 (3.4) | 17 (7.5) |  |
| Other | 46 (8.6) | 22 (9.6) |  |
| DM type |  |  | 0.795 |
| T1DM | 370 (69.5) | 155 (68.3) |  |
| T2DM | 162 (30.5) | 72 (31.7) |  |
| Temperature, ℃ | 37.3 [37.0, 37.7] | 37.3 [37.1, 37.7] | 0.644 |
| HR, beats/min | 108.0 [97.0, 119.0] | 109.0 [98.0, 120.0] | 0.682 |
| RR, breaths/min | 26.0 [22.0, 29.0] | 25.0 [23.0, 29.0] | 0.785 |
| SBP, mmHg | 98.0 [89.0, 108.0] | 97.5 [89.0, 108.0] | 0.684 |
| DBP, mmHg | 46.0 [39.0, 54.0] | 46.0 [38.8, 53.0] | 0.675 |
| Microangiopathy | 197 (37.0) | 69 (30.3) | 0.087 |
| Macroangiopathy | 96 (18.0) | 41 (18.0) | 1.000 |
| Preexisting CKD | 64 (12.0) | 29 (12.7) | 0.885 |
| UTI | 70 (13.2) | 22 (9.6) | 0.184 |
| Pneumonia | 34 (6.4) 14 (6.1) |  |  |
| Liver disease | 44 (8.3) | 16 (7.0) | 0.660 |
| History of Hypertension | 54 (10.2) | 26 (11.4) |  |
| History of CHF | 44 (8.3) | 21 (9.2) | 0.777 |
| Bicarbonate, mEq/L | 14.0 [9.0, 18.0] | 14.0 [8.0, 18.0] | 0.894 |
| WBC, K/uL | 13.5 [9.9, 17.8] | 13.9 [10.1, 17.6] | 0.68 |
| Neutrophil granulocyte, % | 82.6 [74.5, 88.0] | 83.4 [76.2, 89.1] | 0.219 |
| Platelets, K/uL | 303.0 [236.0, 380.0] | 296.0 [234.0, 366.2] | 0.401 |
| Hemoglobin, g/dl | 12.8 [11.4, 14.6] | 12.8 [11.3, 14.3] | 0.486 |
| Sodium, mEq/L | 141.0 [138.0, 144.0] | 141.0 [138.0, 144.0] | 0.397 |
| Chloride, mEq/L | 110.5 [107.0, 115.0] | 111.0 [107.0, 115.0] | 0.569 |
| AG | 21.2 [17.7, 24.7] | 21.1 [17.4, 24.4] | 0.396 |
| Total osmotic pressure | 319.8 [309.2, 335.3] | 319.4 [307.6, 331.7] | 0.099 |
| BUN, mg/dl | 28.0 [17.0, 43.2] | 27.0 [16.0, 40.2] | 0.145 |
| Potassium, mEq/L | 4.3 [3.8, 4.9] | 4.2 [3.8, 4.8] | 0.302 |
| Blood glucose, mg/dl | 310.0 [170.8, 513.0] | 276.0 [154.8, 497.8] | 0.237 |
| SAPS Ⅱ | 27.0 [21.0, 36.0] | 26.0 [19.0, 34.2] | 0.21 |
| OASIS | 25.0 [21.0, 31.0] | 25.0 [21.0, 30.0] | 0.658 |
| SOFA | 2.0 [1.0, 4.0] | 2.0 [1.0, 4.0] | 0.150 |
| GCS | 15.0 [14.0, 15.0] | 15.0 [14.0, 15.0] | 0.344 |
| Infusion volume, ml | 1000.0 [0.0, 2831.2] | 1000.0 [0.0, 2500.0] | 0.242 |
| Urine output, ml | 2095.0 [1400.0, 2950.0] | 2000.0 [1359.0, 3002.5] | 0.664 |
| eGFR | 97.0 [61.4, 121.8] | 100.8 [62.7, 124.0] | 0.505 |
| Use of NaHCO_3_ | 56 (10.5) | 16 (7.0) | 0.168 |
| Mechanical ventilation | 72 (13.5) | 19 (8.3) | 0.057 |
| HLOS, days | 4.6 [2.9, 7.7] | 4.0 [2.7, 6.9] | 0.059 |
| Hospital mortality | 14 (2.6) | 3 (1.3) | 0.421 |
| AKI | 228 (42.9) | 86 (37.7) | 0.216 |

Abbreviations: DM diabetic mellitus, T1DM type 1 diabetic mellitus, T2DM type 2 diabetic mellitus, HR heart rate, RR respiratory rate, SBP systolic blood pressure, DBP diastolic blood pressure, CKD chronic kidney diseases, UTI urinary tract infection, CHF congestive heart failure, WBC white blood cell, AG anion gap, BUN blood urea nitrogen, SAPSII simplified acute physiology score II, OASIS oxford acute severity of illness score, SOFA sequential organ failure assessment, GCS Glasgow coma scale, eGFR estimated glomerular filtration rate, HLOS hospital length of stay, AKI acute kidney injury.
