# Supplementary material for: Nomogram to predict the risk of acute kidney injury in patients with diabetic ketoacidosis: an analysis of the MIMIC-III database
Source: BMC Endocr Disord. 2021 Mar 4;21:37. doi: 10.1186/s12902-021-00696-8 (PMC7931351; doi:10.1186/s12902-021-00696-8)
Supplement: Supplementary file 2 — Additional file 2: Table S2. Univariate logistic regression analysis. [file 12902_2021_696_MOESM2_ESM.docx]

**Table S2** Univariate logistic regression analysis

| Variables | β | OR (95%CI) | P value |
| --- | --- | --- | --- |
| Age, years | 0.01 | 1.01(1.00-1.02) | 0.009 |
| Gender (Female) | 0.02 | 1.03(0.73-1.45) | 0.880 |
| Weight, Kg | 0.01 | 1.01(1.00-1.02) | 0.057 |
| Ethnicity |  |  |  |
| Caucasian | ---- | ---- | ---- |
| African-American | -0.35 | 1.26(0.86-1.85) | 0.228 |
| Hispanic-American | -0.10 | 0.70(0.24-1.86) | 0.494 |
| Other | 68 (8.9) | 0.91(0.47-1.70) | 0.758 |
| DM type (T2DM) | 0.82 | 2.26(1.56-3.30) | <0.001 |
| Temperature, ℃ | 0.33 | 1.39(1.08-1.80) | 0.013 |
| HR, beats/min | 0.00 | 1.00(1.00-1.01) | 1.000 |
| RR, breaths/min | 0.06 | 1.06(1.03-1.09) | <0.001 |
| SBP, mmHg | 0.00 | 1.00(1.00-1.00) | 0.302 |
| DBP, mmHg | -0.02 | 0.98(0.97-1.00) | 0.049 |
| Microangiopathy | 0.48 | 1.61(1.13-2.30) | 0.008 |
| Macroangiopathy | 0.818 | 2.26(1.45-3.57) | <0.001 |
| Preexisting CKD | 1.22 | 3.40 (1.96-6.06) | <0.001 |
| UTI | 0.80 | 2.23(1.34-3.75) | 0.002 |
| Pneumonia | 0.82 | 2.27(1.12-4.75) | 0.024 |
| Liver disease | 0.413 | 1.51(0.81-2.82) | 0.190 |
| History of hypertension | 1.28 | 3.60(1.99-6.81) | <0.001 |
| History of CHF | 1.38 | 3.97(2.05-8.21) | <0.001 |
| Bicarbonate, mEq/L | 0.06 | 1.06(1.03-1.10) | <0.001 |
| WBC, K/uL | 0.01 | 1.01(0.99-1.03) | 0.321 |
| Neutrophil granulocyte, % | -0.01 | 1.67(0.97-1.01) | 0.196 |
| Platelets, K/uL | 0.00 | 1.00(1.00-1.00) | 0.858 |
| Hemoglobin, g/dl | -0.10 | 0.91(0.84-0.98) | 0.015 |
| Sodium, mEq/L | 0.03 | 1.03(1.00-1.06) | 0.028 |
| Chloride, mEq/L | 0.01 | 1.01(0.98-1.03) | 0.621 |
| AG | -0.05 | 0.95(0.92-0.99) | 0.006 |
| Total osmotic pressure | 0.01 | 1.01(1.00-1.01) | 0.141 |
| BUN, mg/dl | 0.01 | 1.01(1.01-1.02) | <0.001 |
| Potassium, mEq/L | -0.11 | 0.90(0.76-1.06) | 0.197 |
| Blood glucose, mg/dl | 0.00 | 1.00(1.00-1.00) | 0.261 |
| GCS | -0.23 | 0.80(0.70-0.89) | <0.001 |
| Infusion volume, ml | -3.4 | 1.00(1.00-1.00) | 0.401 |
| Urine output, ml | 0.00 | 1.00(1.00-1.00) | <0.001 |
| eGFR | 0.00 | 1.00(1.00-1.00) | 0.785 |

Abbreviations: β regression coefficient, OR odds ratios, DM diabetic mellitus, T2DM type 2 diabetic mellitus, HR heart rate, RR respiratory rate, SBP systolic blood pressure, DBP diastolic blood pressure, CKD chronic kidney diseases, UTI urinary tract infection, CHF congestive heart failure, WBC white blood cell, AG anion gap, BUN blood urea nitrogen, GCS Glasgow coma scale, eGFR estimated glomerular filtration rate.
