# Supplementary material for: Nomogram to predict the risk of acute kidney injury in patients with diabetic ketoacidosis: an analysis of the MIMIC-III database
Source: BMC Endocr Disord. 2021 Mar 4;21:37. doi: 10.1186/s12902-021-00696-8 (PMC7931351; doi:10.1186/s12902-021-00696-8)
Supplement: Supplementary file 3 — Additional file 3: SQL code for data extraction. [file 12902_2021_696_MOESM3_ESM.docx]

**Supplementary Materials**

**************************************CODE**************************************

DROP MATERIALIZED VIEW IF EXISTS kdigo_creat CASCADE;

CREATE MATERIALIZED VIEW kdigo_creat as

-- Extract all creatinine values from labevents around patient's ICU stay

with cr as

(

select

ie.icustay_id

, ie.intime, ie.outtime

, le.valuenum as creat

, le.charttime

from icustays ie

left join labevents le

on ie.subject_id = le.subject_id

and le.ITEMID = 50912

and le.VALUENUM is not null

and le.CHARTTIME between (ie.intime - interval '7' day) and (ie.intime + interval '7' day)

)

-- add in the lowest value in the previous 48 hours/7 days

SELECT

cr.icustay_id

, cr.charttime

, cr.creat

, MIN(cr48.creat) AS creat_low_past_48hr

, MIN(cr7.creat) AS creat_low_past_7day

FROM cr

-- add in all creatinine values in the last 48 hours

LEFT JOIN cr cr48

ON cr.icustay_id = cr48.icustay_id

AND cr48.charttime < cr.charttime

AND cr48.charttime >= (cr.charttime - INTERVAL '48' HOUR)

-- add in all creatinine values in the last 7 days hours

LEFT JOIN cr cr7

ON cr.icustay_id = cr7.icustay_id

AND cr7.charttime < cr.charttime

AND cr7.charttime >= (cr.charttime - INTERVAL '7' DAY)

GROUP BY cr.icustay_id, cr.charttime, cr.creat

ORDER BY cr.icustay_id, cr.charttime, cr.creat;

-- This query checks if the patient had AKI according to KDIGO.

-- AKI is calculated every time a creatinine or urine output measurement occurs.

-- Baseline creatinine is defined as the lowest creatinine in the past 7 days.

DROP MATERIALIZED VIEW IF EXISTS kdigo_stages CASCADE;

CREATE MATERIALIZED VIEW kdigo_stages AS

-- get creatinine stages

with cr_stg AS

(

SELECT

cr.icustay_id

, cr.charttime

, cr.creat

, case

-- 3x baseline

when cr.creat >= (cr.creat_low_past_7day*3.0) then 3

-- *OR* cr >= 4.0 with associated increase

when cr.creat >= 4

-- For patients reaching Stage 3 by SCr >4.0 mg/dl

-- require that the patient first achieve ... acute increase >= 0.3 within 48 hr

-- *or* an increase of >= 1.5 times baseline

and (cr.creat_low_past_48hr <= 3.7 OR cr.creat >= (1.5*cr.creat_low_past_7day))

then 3

-- TODO: initiation of RRT

when cr.creat >= (cr.creat_low_past_7day*2.0) then 2

when cr.creat >= (cr.creat_low_past_48hr+0.3) then 1

when cr.creat >= (cr.creat_low_past_7day*1.5) then 1

else 0 end as aki_stage_creat

FROM kdigo_creat cr

)

-- stages for UO / creat

, uo_stg as

(

select

uo.icustay_id

, uo.charttime

, uo.weight

, uo.uo_rt_6hr

, uo.uo_rt_12hr

, uo.uo_rt_24hr

-- AKI stages according to urine output

, CASE

WHEN uo.uo_rt_6hr IS NULL THEN NULL

-- require patient to be in ICU for at least 6 hours to stage UO

WHEN uo.charttime <= ie.intime + interval '6' hour THEN 0

-- require the UO rate to be calculated over half the period

-- i.e. for uo rate over 24 hours, require documentation at least 12 hr apart

WHEN uo.uo_tm_24hr >= 11 AND uo.uo_rt_24hr < 0.3 THEN 3

WHEN uo.uo_tm_12hr >= 5 AND uo.uo_rt_12hr = 0 THEN 3

WHEN uo.uo_tm_12hr >= 5 AND uo.uo_rt_12hr < 0.5 THEN 2

WHEN uo.uo_tm_6hr >= 2 AND uo.uo_rt_6hr < 0.5 THEN 1

ELSE 0 END AS aki_stage_uo

from kdigo_uo uo

INNER JOIN icustays ie

ON uo.icustay_id = ie.icustay_id

)

-- get all charttimes documented

, tm_stg AS

(

SELECT

icustay_id, charttime

FROM cr_stg

UNION

SELECT

icustay_id, charttime

FROM uo_stg

)

select

ie.icustay_id

, tm.charttime

, cr.creat

, cr.aki_stage_creat

, uo.uo_rt_6hr

, uo.uo_rt_12hr

, uo.uo_rt_24hr

, uo.aki_stage_uo

-- Classify AKI using both creatinine/urine output criteria

, GREATEST(cr.aki_stage_creat, uo.aki_stage_uo) AS aki_stage

FROM icustays ie

-- get all possible charttimes as listed in tm_stg

LEFT JOIN tm_stg tm

ON ie.icustay_id = tm.icustay_id

LEFT JOIN cr_stg cr

ON ie.icustay_id = cr.icustay_id

AND tm.charttime = cr.charttime

LEFT JOIN uo_stg uo

ON ie.icustay_id = uo.icustay_id

AND tm.charttime = uo.charttime

order by ie.icustay_id, tm.charttime;

-- This query checks if the patient had AKI during the first 7 days of their ICU

-- stay according to the KDIGO guideline.

-- https://kdigo.org/wp-content/uploads/2016/10/KDIGO-2012-AKI-Guideline-English.pdf

DROP MATERIALIZED VIEW IF EXISTS kdigo_stages_7day;

CREATE MATERIALIZED VIEW kdigo_stages_7day AS

-- get the worst staging of creatinine in the first 48 hours

WITH cr_aki AS

(

SELECT

k.icustay_id

, k.charttime

, k.creat

, k.aki_stage_creat

, ROW_NUMBER() OVER (PARTITION BY k.icustay_id ORDER BY k.aki_stage_creat DESC, k.creat DESC) AS rn

FROM icustays ie

INNER JOIN kdigo_stages k

ON ie.icustay_id = k.icustay_id

WHERE k.charttime > (ie.intime - interval '6' hour)

AND k.charttime <= (ie.intime + interval '7' day)

AND k.aki_stage_creat IS NOT NULL

)

-- get the worst staging of urine output in the first 48 hours

, uo_aki AS

(

SELECT

k.icustay_id

, k.charttime

, k.uo_rt_6hr, k.uo_rt_12hr, k.uo_rt_24hr

, k.aki_stage_uo

, ROW_NUMBER() OVER

(

PARTITION BY k.icustay_id

ORDER BY k.aki_stage_uo DESC, k.uo_rt_24hr DESC, k.uo_rt_12hr DESC, k.uo_rt_6hr DESC

) AS rn

FROM icustays ie

INNER JOIN kdigo_stages k

ON ie.icustay_id = k.icustay_id

WHERE k.charttime > (ie.intime - interval '6' hour)

AND k.charttime <= (ie.intime + interval '7' day)

AND k.aki_stage_uo IS NOT NULL

)

-- final table is aki_stage, include worst cr/uo for convenience

select

ie.icustay_id

, cr.charttime as charttime_creat

, cr.creat

, cr.aki_stage_creat

, uo.charttime as charttime_uo

, uo.uo_rt_6hr

, uo.uo_rt_12hr

, uo.uo_rt_24hr

, uo.aki_stage_uo

-- Classify AKI using both creatinine/urine output criteria

, GREATEST(cr.aki_stage_creat,uo.aki_stage_uo) AS aki_stage_7day

, CASE WHEN GREATEST(cr.aki_stage_creat, uo.aki_stage_uo) > 0 THEN 1 ELSE 0 END AS aki_7day

FROM icustays ie

LEFT JOIN cr_aki cr

ON ie.icustay_id = cr.icustay_id

AND cr.rn = 1

LEFT JOIN uo_aki uo

ON ie.icustay_id = uo.icustay_id

AND uo.rn = 1

order by ie.icustay_id;

*******************************************************************************
